# Pleomorphic Adenoma of the Palate: A Case Report

**DOI:** 10.7759/cureus.116762

**Published:** 2026-09-23

**Authors:** Sreesha S, Roopesh U, Joseph Edward, M Devanarayanan Potti, Anjali Anandan Unnithan, Ajimsha SB

**Affiliations:** 1 Department of Dental Sciences, Azeezia College of Dental Sciences and Research, Kollam, IND; 2 Department of Dental Sciences, Kerala University of Health Sciences, Thrissur, IND

**Keywords:** hard palate, minor salivary glands, pleomorphic adenoma, salivary gland neoplasm, surgical excision

## Abstract

Pleomorphic adenoma is a benign mixed salivary gland neoplasm that most commonly arises in the parotid gland, while involvement of the minor salivary glands is uncommon. This case report describes a 46-year-old woman who presented with a painless, gradually enlarging swelling on the right posterolateral aspect of the palate for one year. Intraoral examination revealed a solitary, firm, immobile, non-tender, non-pulsatile swelling measuring approximately 3 × 2 cm, covered by smooth and intact mucosa. Histopathological examination of an incisional biopsy demonstrated epithelial proliferation arranged in sheets, nests, cords, and duct-like structures, together with plasmacytoid cells and hyalinized, myxoid, and chondroid-like stromal areas, confirming pleomorphic adenoma. The lesion was completely excised under local anesthesia along with the associated mucoperiosteum and eroded palatal bone, and the wound was closed with 3-0 silk sutures. The patient experienced no immediate postoperative complications and showed no clinical recurrence during six months of follow-up. This case emphasizes the importance of histopathological confirmation, complete surgical excision, and continued postoperative surveillance in the management of pleomorphic adenoma of the hard palate.

## Introduction

Pleomorphic adenoma is a benign salivary gland tumor composed of epithelial, myoepithelial, and variable stromal components. Although it most commonly arises in the parotid gland, it can also develop from the minor salivary glands of the oral cavity, with the palate being the most frequently affected intraoral site because of its high concentration of minor salivary glands [1]. Long-standing or recurrent pleomorphic adenomas have a potential for malignant transformation into carcinoma ex pleomorphic adenoma, emphasizing the importance of timely diagnosis and complete treatment [2].

Clinically, palatal pleomorphic adenoma usually presents as a painless, slowly enlarging, firm swelling beneath intact palatal mucosa [3]. Because this appearance can overlap with that of other benign and malignant palatal lesions, histopathological examination is required for definitive diagnosis [4]. Pleomorphic adenomas arising outside the major salivary glands are relatively uncommon and therefore require careful clinical and pathological evaluation [5].

This report describes a 46-year-old woman with a painless, gradually enlarging pleomorphic adenoma of the right posterior hard palate that had been present for one year. Histopathological examination confirmed the diagnosis, and complete surgical excision included the associated mucoperiosteum and clinically eroded palatal bone. The presence of palatal bone erosion despite an otherwise painless, slowly progressive clinical presentation adds clinical relevance to this case and highlights the importance of careful assessment, histopathological confirmation, adequate surgical excision, and postoperative surveillance.

## Case presentation

A 46-year-old female patient presented to the maxillofacial center with a non-painful swelling of the palate that she had noticed about one year ago. This swelling had grown slowly to this size without any bouts of sudden growth. No numbness, dysphagia, stridor, speech difficulties, or mastication difficulties were reported. No fever, trauma, or previous similar swelling in the body was noted. She has no known systemic disease and no known harmful habits. She noted that she had had a tooth pulled in the past, but there was no documentation of the extracted tooth, indication, timing, or temporal relationship of the tooth extraction to the development of the palatal swelling. Thus, it was not possible to conclude that there was a causal relationship between the previous extraction and the present lesion.

A moderately built conscious woman with a normal gait was evident on general physical examination. Her blood pressure, temperature, and pulse were normal. The facial examination was normal, and there was no facial asymmetry or regional lymphadenopathy. The intraoral examination revealed a single ovoid mass on the right posterolateral part of the posterior palate measuring about 3 × 2 cm. The lesion was confined between the region of tooth 14 anteriorly and the region of tooth 17 posteriorly, and its inner edge was close to the midline of the hard palate, with its outer edge reaching the distal surface of the region of tooth 17. The overlying mucosa was smooth, healthy, and without any secondary changes or ulceration. On palpation, the lesion was well defined, firm, non-tender, non-pulsatile, immobile, and apparently unilocular. The overlying mucosa was stretched and was not separable from the swelling. The clinical presentation of the palatal swelling is illustrated in Figure 1.

**Figure 1:**
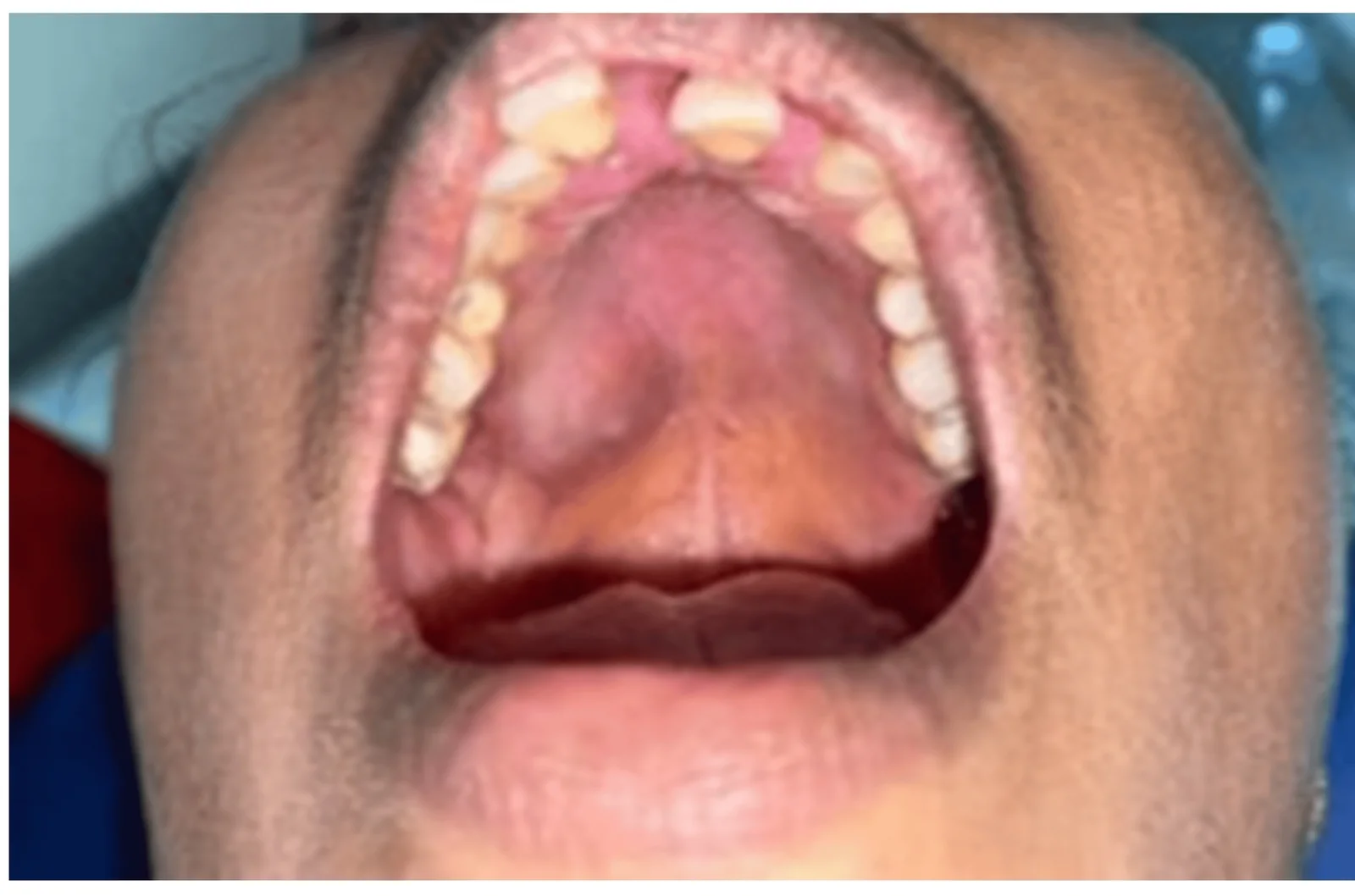
Preoperative intraoral appearance of the palatal lesion

A single, distinct mass was seen in the right posterolateral area of the hard palate. A biopsy was taken from the back of the palate and sent to the histopathology laboratory. The epithelial cell proliferation was seen in sheets, small nests, cords, and ducts when examined under the microscope. There were also a few plasmacytoid cells. The mesenchymal component was hyalinized and had myxoid areas with vacuolar degeneration, like chondroid tissue. Intracapsular and perivascular tumor cells were reported. This diagnosis was made based on these findings: pleomorphic adenoma. The related pathological changes are depicted in Figure 2.

**Figure 2:**
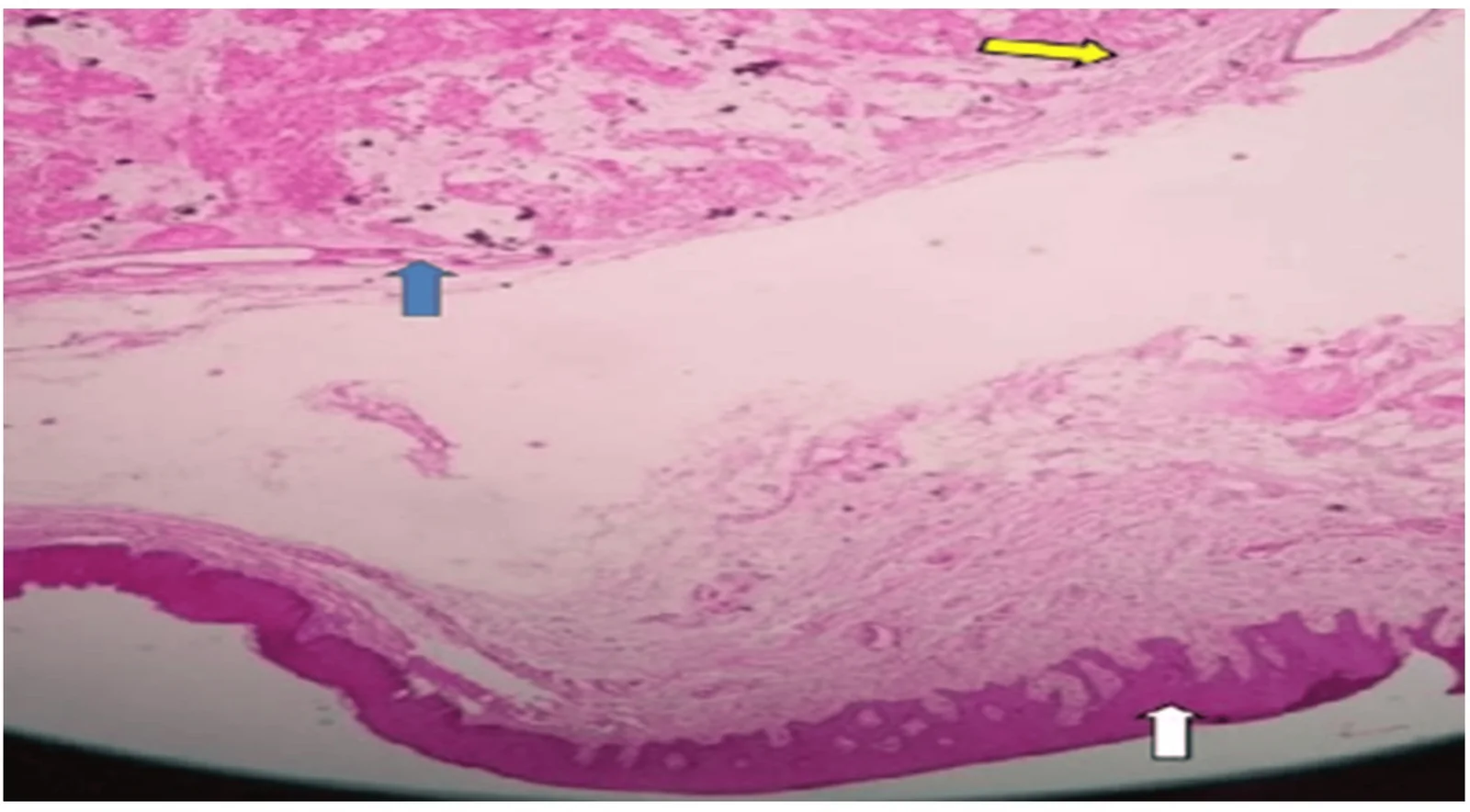
Histopathological features of pleomorphic adenoma Histopathological image showing islands of myoepithelial cells (blue arrow), mesenchymal connective tissue (yellow arrow), and parakeratinized stratified squamous epithelium (white arrow) (hematoxylin and eosin stain, ×100 magnification).

Upon confirmation of diagnosis, surgical excision was planned. Local anesthetic was used, and the patient was prepared and draped. A circumferential incision was made around the palatal lesion using a number 15 Bard-Parker blade. The lesion was completely excised together with the associated mucoperiosteum and clinically eroded palatal bone, with the dissection extending into clinically normal surrounding tissue. Grossly, the lesion appeared encapsulated. Preoperative cone beam computed tomography (CBCT)/computed tomography (CT) imaging was not performed because the lesion was small and clinically localized, without findings suggesting underlying bone or adjacent structure involvement requiring cross-sectional imaging. The available histopathological examination confirmed pleomorphic adenoma; however, microscopic surgical margin status and detailed histopathological assessment of capsular integrity were not documented in the available clinical record. The immediate operative appearance is illustrated in Figure 3.

**Figure 3:**
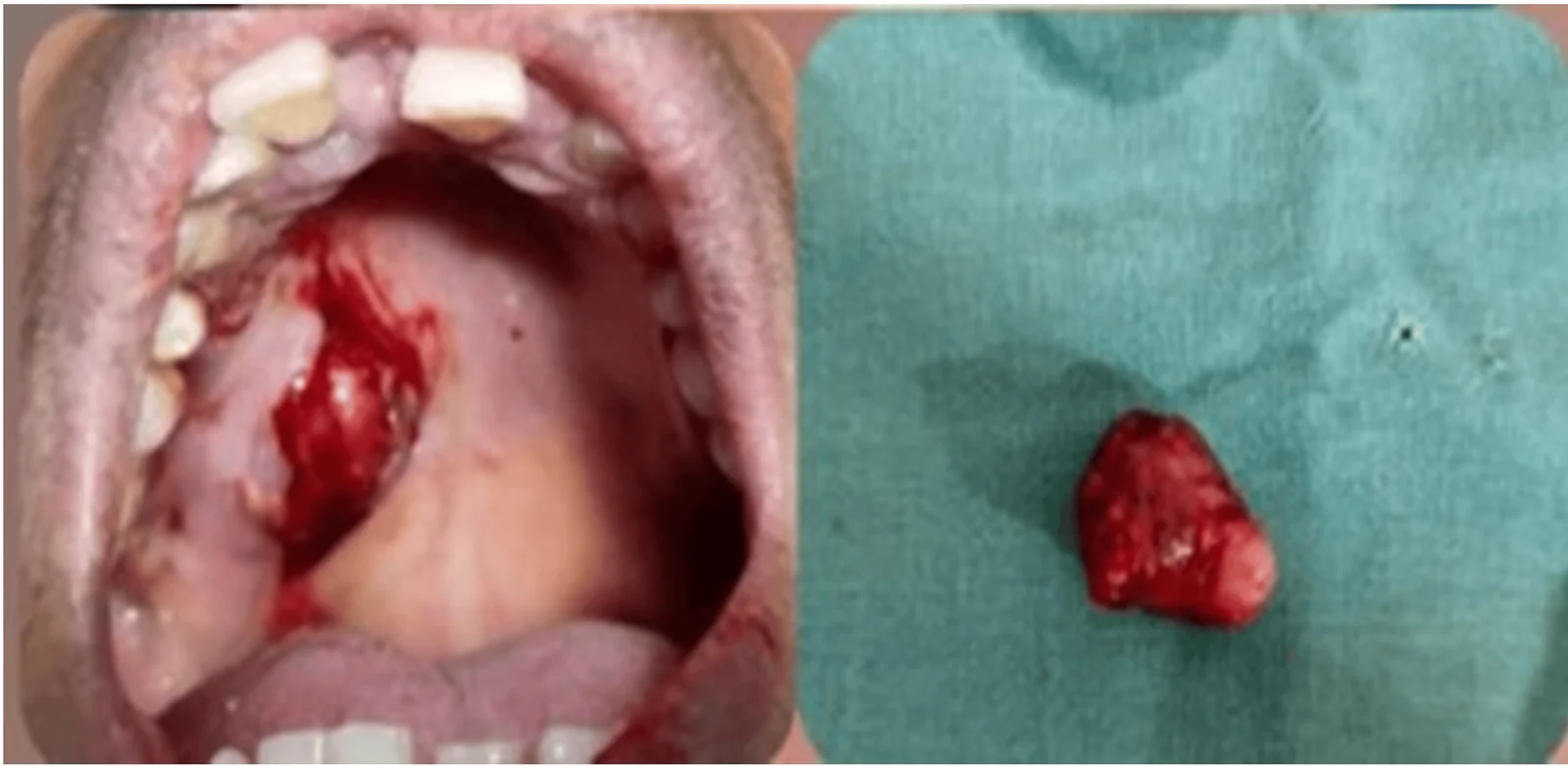
Surgical excision of the palatal lesion

Hemostasis was established, and 3-0 silk sutures were used for surgical closure. Postoperatively, the patient received antibiotics and analgesics. There was no report of any immediate postoperative complications. The site of the operation after removal of the palatal mass and the excised tumor specimen is presented in Figure 4.

**Figure 4:**
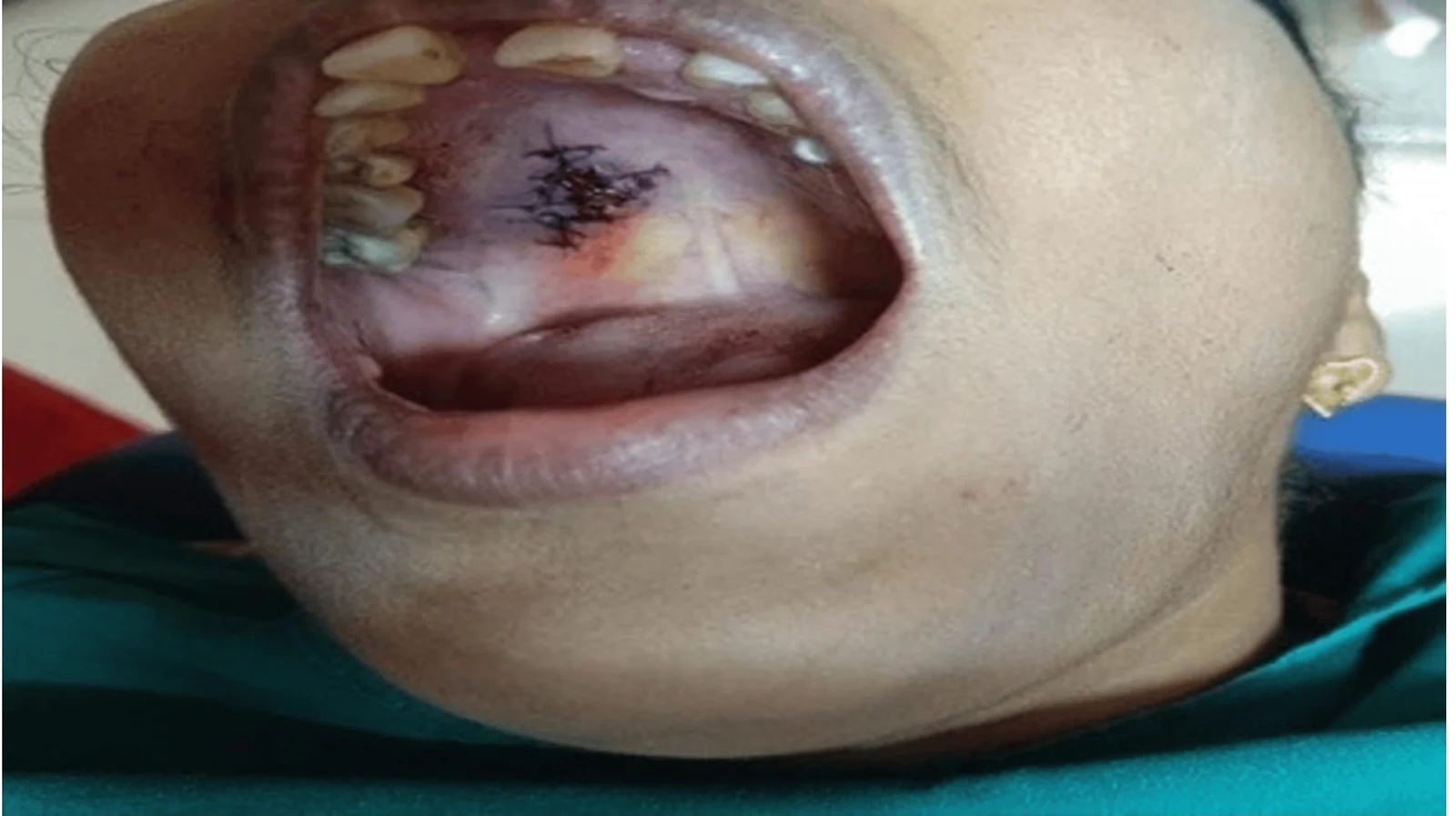
Immediate postoperative appearance

The patient continued clinical follow-up for six months. During this period, no postoperative complications were documented, and no clinical recurrence was observed at the final six-month assessment. A detailed visit-by-visit follow-up schedule, timing of suture removal, visit-specific wound healing findings, and postoperative imaging details were not available in the clinical record. Long-term follow-up was advised because recurrence of pleomorphic adenoma may occur after a prolonged interval. The clinical course is summarized in Table 1.

**Table 1:** Clinical course, treatment, and follow-up

Time point	Clinical event	Key finding or outcome
One year before presentation	Patient first noticed a palatal swelling	Painless lesion with gradual enlargement
At presentation	Clinical examination performed	Solitary 3 × 2 cm firm, immobile swelling on the right posterolateral palate; no ulceration, tenderness, pulsation, facial asymmetry, or lymphadenopathy
Diagnostic evaluation	Incisional biopsy obtained from the posterior palatal region	Histopathology showed epithelial and myoepithelial proliferation with myxoid, hyalinized, and chondroid-like stromal areas, confirming pleomorphic adenoma
Definitive treatment	Surgical excision performed under local anesthesia	Lesion excised with the associated mucoperiosteum and clinically eroded palatal bone; dissection extended into clinically normal surrounding tissue
Immediate postoperative period	Hemostasis and wound closure completed	Surgical site closed with 3-0 silk sutures; antibiotics and analgesics administered; no immediate postoperative complications were documented
Six-month follow-up	Periodic clinical assessment	No postoperative complications or clinical evidence of recurrence at the final six-month assessment

## Discussion

The present case was that of a 46-year-old woman who had had a swelling on the right posterolateral aspect of the palate for one year that had gradually increased over time and had been painless. The lesion was located at the apex of the hard palate; was firm, non-pulsatile, immobile, and non-tender; and had a smooth, healthy mucous membrane overlying the lesion; it measured about 3 × 2 cm in size. There was no facial asymmetry, no lymphadenopathy, no difficulty with swallowing or speaking, no numbness, and no associated symptoms. Histopathological examination revealed sheets, nests, cords, and duct-like arrangement of the epithelial proliferation, plasmacytoid cells, and hyalinized, myxoid, and chondroid-like stromal areas. The results of these findings confirmed the diagnosis of pleomorphic adenoma. The lesion was completely excised with removal of the associated mucoperiosteum and palatal bone that had been eroded. During the six-month follow-up period, no postoperative complications or clinical recurrence was reported. Pleomorphic adenoma of the minor salivary glands is rare but occurs most commonly on the palate due to the relatively high density of minor salivary gland tissue in the palate. Palatal lesions are most common in adulthood and are slightly more common in women than in men [6]. As such, the age and sex of the patient were consistent with the commonly reported age and sex distribution. Pleomorphic adenoma of the palate has also been previously reported as a painless, slow-growing, firm, submucosal swelling, which is commonly found on the posterolateral aspect of the hard or soft palate [7]. In the present case, these clinical manifestations, which are generally reported, were observed: smooth mucosal surface, no ulceration, gradual enlargement, and no neurological or functional symptoms.

In the present case, the lesion was 3 × 2 cm in size, a larger size than the frequently reported smaller palatal lesions. Larger lesions can be asymptomatic if growth is slow but, at some point, can be a problem with speech, chewing, or swallowing [8]. The present patient had no functional problem, although the lesion was large. The fact that the swelling was immobile also supported its palatal location because other benign lesions, which adhere tightly to the mucosa and underlying periosteum of the hard palate, can appear immobile [9]. Histopathological examination is still necessary as the clinical characteristics of pleomorphic adenoma may also be like other benign and malignant lesions of the palate. Characteristic micro pattern consists of epithelial and myoepithelial cells in variable configuration embedded in myxoid, hyalinized, or chondroid-like stromal tissue [7]. The plasmacytoid myoepithelial cells have also been reported in pleomorphic adenomas of minor salivary glands [8]. The epithelial sheets, nests, cords, and duct-like structures, plasmacytoid cells, and mixed stromal components documented in the present case were similar to the features previously reported. A few differential diagnoses must be considered when a swelling is seen in the palate: mucocele, hematoma, necrotizing sialometaplasia, palatal abscess, neurofibroma, neuroma, mucoepidermoid carcinoma, adenoid cystic carcinoma, and polymorphous adenocarcinoma [8]. Rapid growth, spontaneous ulceration, pain, paresthesia, indistinct margins, or regional lymphadenopathy can heighten the suspicion of malignancy. All these findings were not reported in the present patient. A histopathological evaluation was thus necessary to confirm the diagnosis and to rule out clinically similar lesions [9].

Pleomorphic adenoma of the palate is accepted to be treated with complete surgical removal [10]. The lesion and any clinically involved tissue should be excised with care to not disrupt the tumor capsule. In this instance, the lesion was excised together with the associated mucoperiosteum and eroded palatal bone, and the boundaries were set in the surrounding clinically healthy tissue. Similar cases of pleomorphic adenoma of the hard palate have also been managed by complete surgical excision [11]. Incomplete resection, microscopic extensions, disruption of the capsule, or intraoperative release of the tumor are associated with the risk of local recurrence [10]. Pleomorphic adenoma must be completely removed because it may have an incomplete capsule or microscopic projections into the surrounding tissue (pseudopods). There may also be a risk of malignant change in long-standing or recurrent tumors [12]. There was no clinical recurrence in the present patient during the six-month follow-up period; however, this duration is too short to exclude delayed recurrence. Long-term clinical monitoring is therefore required. In this case, the clinical presentation was consistent with the characteristic features of palatal pleomorphic adenoma, particularly a painless, slowly enlarging palatal swelling. Appropriate management entails careful clinical examination, histopathological confirmation, complete surgical removal, and long-term follow-up.

There are a few limitations of this case report. The follow-up period was limited to six months, which is insufficient to exclude delayed recurrence of pleomorphic adenoma. A detailed postoperative follow-up schedule and visit-specific wound healing findings were not available in the clinical record. Detailed information regarding postoperative medications, including the name, dosage, route, frequency, and duration, was also unavailable. The timing and indication of the previous tooth extraction, as well as its temporal relationship with the palatal swelling, could not be established. Preoperative CBCT/CT imaging was not performed, and therefore, no cross-sectional imaging findings were available. Microscopic surgical margin status and detailed histopathological assessment of capsular integrity were also not documented in the available clinical record. The clinical photographs did not include a scale reference or periodontal probe; therefore, the reported lesion dimensions were based on clinical examination rather than estimation from the images. The excised specimen photograph also lacked a ruler or scale reference, limiting direct visual assessment of specimen dimensions. These limitations restrict assessment of the full extent of the lesion and the long-term risk of recurrence. Future reports should include appropriate preoperative imaging when clinically indicated, complete pathological assessment of the excised specimen and surgical margins, standardized clinical and specimen photography with an appropriate scale reference, and detailed documentation of perioperative management. Long-term clinical surveillance remains important because recurrence may occur several years after excision. Regular clinical follow-up, supplemented by imaging when clinically indicated, may assist in detecting persistent or recurrent disease. Larger multicenter case series are also needed to evaluate recurrence predictors, appropriate surgical margins, wound management strategies, and long-term functional outcomes.

## Conclusions

This case report describes a pleomorphic adenoma of the hard palate in a 46-year-old woman who presented with a one-year history of a painless, progressively enlarging palatal swelling. Histopathological examination confirmed the diagnosis, and the lesion was managed by complete surgical excision together with the associated mucoperiosteum and clinically eroded palatal bone. No postoperative complications or clinical recurrence were observed during the six-month follow-up period, indicating a favorable short-term outcome. This case highlights the importance of systematic evaluation of persistent palatal swellings because benign and malignant minor salivary gland lesions may have similar clinical presentations. Histopathological confirmation remains essential for definitive diagnosis, while complete surgical excision and long-term surveillance are important because delayed recurrence may occur. Given the limited follow-up duration and the absence of documented microscopic margin status and detailed capsular assessment, longer-term monitoring is warranted.
